# Supplementary material for: Clonal Dissemination of Pandrug-Resistant Klebsiella pneumoniae ST392KL27 in a Tertiary Care Hospital in Mexico
Source: Int J Mol Sci. 2025 Aug 20;26(16):8047. doi: 10.3390/ijms26168047 (PMC12386949; doi:10.3390/ijms26168047)
Supplement: Supplementary file 1 [file ijms-26-08047-s001.zip › ijms-3802346-supplementary.pdf]

International Journal of
Molecular Sciences

MDPI

Table S1. Assembly quality of the *Klebsiella pneumoniae* genomes.

	Genome size	Genome fraction[1]	Number of contigs[2]	N50[3]	GC%	Duplication ratio[4]
HJM-EMC466	5,57 Mbp	85.78%	265	58,956	57.23	1.002
HJM-NEE391	5,58 Mbp	85.76%	221	86,389	57.18	1.002
HJM-ERG332	5.51 Mbp	84.93%	391	50,484	57.29	1.003
HJM-ICO381	5.60 Mbp	85.57%	339	71,513	57.17	1.003
HJM-CBA208	5.59 Mbp	85.86%	182	79,525	57.18	1.001
HJM-CCQ423	5.55 Mbp	85.47%	170	81,443	57.2	1.001

[1]Number of aligned bases in the reference, divided by the genome size. A base in the reference genome is counted as aligned if at least one contig has at least one alignment to this base.[2]Total number of contigs in the assembly. [3]The length for which the collection of all contigs of that length or longer covers at least 50% of the assembly length.[4]Ratio of aligned bases in the assembly to the aligned bases in the reference.
